# The Potential of Donor T-Cell Repertoires in Neoantigen-Targeted Cancer Immunotherapy

**DOI:** 10.3389/fimmu.2017.01718

**Published:** 2017-12-11

**Authors:** Terhi Karpanen, Johanna Olweus

**Affiliations:** ^1^Department of Cancer Immunology, Institute for Cancer Research, Oslo University Hospital Radiumhospitalet, and K.G. Jebsen Center for Cancer Immunotherapy, University of Oslo, Oslo, Norway

**Keywords:** neoantigen, immunotherapy, T cell, donor, allogeneic hematopoietic stem cell transplantation, donor lymphocyte infusion, minor histocompatibility antigen, graft versus tumor effect

## Abstract

T cells can recognize peptides encoded by mutated genes, but analysis of tumor-infiltrating lymphocytes suggests that very few neoantigens spontaneously elicit T-cell responses. This may be an important reason why immune checkpoint inhibitors are mainly effective in tumors with a high mutational burden. Reasons for clinically insufficient responses to neoantigens might be inefficient priming, inhibition, or deletion of the cognate T cells. Responses can be dramatically improved by cancer immunotherapy such as checkpoint inhibition, but often with temporary effects. By contrast, T cells from human leukocyte antigen (HLA)-matched donors can cure diseases such as chronic myeloid leukemia. The therapeutic effect is mediated by donor T cells recognizing polymorphic peptides for which the donor and patient are disparate, presented on self-HLA. Donor T-cell repertoires are unbiased by the immunosuppressive environment of the tumor. A recent study demonstrated that T cells from healthy individuals are able to respond to neoantigens that are ignored by tumor-infiltrating T cells of melanoma patients. In this review, we discuss possible reasons why neoantigens escape host T cells and how these limitations may be overcome by utilization of donor-derived T-cell repertoires to facilitate rational design of neoantigen-targeted immunotherapy.

## Introduction

Neoantigens derived from somatic mutations in cancer cells and recognized as foreign by T cells are arising as the most attractive targets of cancer immunotherapy. They are expressed exclusively in malignant cells, making them truly tumor specific, and the T-cell repertoire recognizing them is not affected by central tolerance mechanisms. Recent studies have demonstrated a correlation between the clinical benefit of cancer immunotherapies such as checkpoint inhibition, with mismatch-repair deficiency, burden of somatic nonsynonymous mutations and neoantigen load (1–5), and neoantigen-reactive T cells have been detected in many tumors (Table 1).

**Table 1:** Only a small fraction of candidate neoantigens elicits spontaneous responses in the cancer patient’s autologous T-cell repertoire.

Tumor type (source of neoantigen-reactive T cells)	Total number of nonsynonymous mutations/number of patients analyzed	Mutations encoding neoepitopes/mutations screened (immunogenic peptides identified/peptides screened^a^)	% Mutations encoding neoepitopes (% immunogenic peptides^a^)	Test method (pipeline for preselection)	Reference
**Autologous, spontaneous**					

Melanoma (TIL)	1,084/1	2/448^a^	0.4^a^	pMHC (RNAseq, NetChopCterm3.0, NetMHC3.2)	(6)
Melanoma (TIL)	1,116/3	7/191	3.7	IFNg (NetMHCpan2.4)	(7)
		11/227^a^	4.8^a^		
Melanoma (TIL)	np/2	2/288	0.7	TM and IFNg (none)	(8)
Ovarian cancer (TAL)	93/3	1/79	1.3	ELISpot (NetMHCpan2.4)	(9)
Gastrointestinal cancers (TIL)	773^b^/10	18/1,452	1.2	TM and ELISpot/CD137 (none)	(10, 11)
Melanoma (FTD and TIL)	19,597/8	10/369^a^	2.7^a^	pMHC (RNAseq, IEDB)	(12)
Melanoma [TIL or PBMC-derived infusion product]	2,386/5	8/1,543	0.5	ELISA (RNAseq)	(13)
Melanoma (CD8^+^PD-1^+^ PBMC)	1,479/4	7/691	1.0	TM and ELISpot/CD137 (RNAseq)	(14)
Melanoma (TIL)	1,100/3	2/201	1.0	pMHC (RNAseq, NetChopCterm3.0, NetMHC3.2, or NetMHCpan2.0)	(15)
		2/391^a^	0.5^a^		
Melanoma (TIL)	>4,000/1	10/720	1.4	TM and IFNq ELISA (RNAseq, IEDB)	(16)
Melanoma (TIL)	np/4^c^	12/675	1.8	TM and CD137 (IEDB)	(17)
NSCLC (TIL and T cells from adjacent normal tissue)	np/2	3/642^a^	0.5^a^	pMHC (NetMHCpan2.8)	(18)
Melanoma (TIL)	1,019/1	1/2	50.0	ELISA (MS/MS)	(19)
NSCLC (TIL and PBMC)	np/2	9/705^a^	1.3^a^	DNA-barcoded pMHC staining (NetMHCpan2.8)	(20)

**Autologous, therapeutically induced**					

NSCLC (PBL after anti-PD-1)	324/1	1/99	1.0	pHMC (NetMHC3.4)	(3)
		1/148^a^	0.7^a^		
Colorectal cancer (PBMC after anti-PD-1)	1,477^b^/1	3/15	20.0	ELISpot (ImmunoSelect-R)	(5)
		3/15^a^	20.0^a^		
Melanoma (PBMC after anti-CTLA-4)	2,329/1	2/8	25.0	ELISpot (LC-MS/MS)	(21)
		2/8^a^	25.0^a^		
Melanoma (PBMC after peptide loaded dendritic cell vaccination)	1,099^d^/3	9/21	42.9	pMHC (MS/MS)	(22)
		9/21^a^	42.9^a^		
Lung squamous cell carcinoma (PBMC after peptide vaccination)	93/1	4/5	80.0	ELISpot (NetMHC3.4)	(23)
		6/11^a^	54.5^a^		
Melanoma (PBMC after peptide vaccination)	4,729/6	15/91	16.5 (CD8)	ELISpot (RNAseq, NetMHCpan2.4)	(24)
		58/97	59.8 (CD4)		

**Donor derived**					

CLL (PBMC after alloHSCT)	51/2	3/25	12.0	ELISpot (NetMHCpan2.4)	(25)
		3/48^a^	6.3^a^		
Melanoma (PBMC from healthy individuals)	6,413/5	4/11	36.4	ELISpot (LC-MS/MS)	(21)
		4/11^a^	36.4^a^		
Melanoma (PBMC from healthy individuals)	1,100/3	10/45	22.2	pMHC (RNAseq, NetMHC4.0)	(15)
		11/57^a^	19.3^a^		

*Summary of studies in which mutations encoding candidate neoantigens were identified by whole-exome sequencing and systematically screened for recognition by T-cells. For each study, the table indicates the number of mutations encoding neoepitopes (immunogenic peptides) that were identified among the number of mutations screened, and/or, when indicated by an ^a^, the number of immunogenic peptides (neoepitopes) identified among the total number of peptides screened (multiple candidate peptides can be screened for a single mutation)*.

*^b^Total number of mutations (when number of nonsynonymous mutations was not reported)*.

*^c^Only the four patients for which tandem minigene constructs were available are included*.

*^d^Nonsynonymous mutations in lymph node or axilla*.

*np, information not provided; TIL, tumor-infiltrating lymphocytes; TAL, tumor-associated lymphocytes; FTD, fresh tumor digest; PBMC, peripheral blood mononuclear cells; NSCLC, non-small-cell lung carcinoma; PBL, peripheral blood lymphocytes; CLL, chronic lymphocytic leukemia; TM, tandem minigene; MS/MS, tandem mass spectrometry; LC-MS/MS, liquid chromatography-tandem mass spectrometry; pMHC, peptide-major histocompatibility complex molecule multimers; IEDB, The Immune Epitope Database and Analysis Resource*.

Many tumors harbor a large number of mutations that potentially can give rise to neoepitopes (26). All mutations leading to single amino acid substitutions, reading-frame alterations, splice variants, inversions, fusions, and aberrant posttranslational modifications, have the potential to generate neoantigens. For a neoantigen to be immunogenic, it has to be expressed at sufficient levels, have the correct subcellular localization to enter proteasomes, be efficiently processed and transported to the endoplasmic reticulum, be loaded on human leukocyte antigen (HLA)-molecules with high enough affinity, form a complex with HLA with sufficient stability, and be efficiently recognized by the patient T-cell repertoire. Current sequencing techniques and computational sequence analysis tools enable rapid calling of somatic mutations from individual tumors. However, identification of verified neoepitopes from the large pool of candidate peptides remains a major challenge on the way to efficiently exploit each patient’s unique set of neoantigens for targeted immunotherapy. The vast majority of neoantigens are personal and few shared neoantigens have been found to be immunogenic, highlighting the need for efficient strategies for fast identification of personal neoepitopes. Prediction methods for HLA-binding affinity, at least for the most frequent HLA class I alleles, can narrow down the number of candidate neoepitopes, but are insufficient in predicting proteasomal processing, transport of the peptides, stability of the peptide–HLA complexes, and recognition by T cells. This makes the identification of clinically relevant neoepitopes with therapeutic potential challenging.

In this review, we discuss possible reasons for the insufficiency of the patient’s T cells to respond to neoantigens and experience gained from the use of donor T cells in the treatment of hematological malignancies. We will also focus on recent insights gained from the use of T-cell repertoires from healthy individuals to identify immunogenic neoantigens and present possibilities these insights open for the efficient clinical exploitation of personal neoantigens.

## The Autologous T-Cell Repertoire of the Patient Frequently Fails to Control Cancer Progression

The potential of neoantigen-specific T cells to induce cancer regression has unequivocally been demonstrated. Infusion of selected patient-derived, neoantigen-reactive T cells were shown to induce objective clinical responses in a patient with epithelial cancer, treated with enriched CD4 T cells recognizing a mutant erbb2 interacting protein (ERBB2IP)-derived peptide (10), and in a colorectal cancer patient treated with a cytotoxic T-cell pool consisting of four different clonotypes specific for KRAS G12D-derived peptides presented by HLA-C*08:02 (27). Analysis of T cells derived from tumor-infiltrating lymphocytes (TIL) or peripheral blood has, however, shown that the frequency of neoantigens that can elicit such responses is low: Only about 1.2% of mutations with neoantigenic potential are spontaneously recognized in patients with melanoma, gastrointestinal, lung, and ovarian cancers. T cells responsive to 68 of the 5,842 candidate neoantigens screened were found in 36 patients included in 11 studies (Table 1; 7–11, 13–17, 19). Similarly, very few mutations were identified that evoke an immune response in patients treated with checkpoint inhibition (3). A multitude of mechanisms might collectively be responsible for this. Tumors can actively suppress existing T-cell responses, extensively reviewed elsewhere (28). Strategies include secretion of immunosuppressive cytokines, attraction of regulatory T cells or myeloid-derived suppressor cells, upregulation of the expression of inhibitory molecules, such as immune checkpoint receptors on T cells (29) and their ligands in tumor cells, loss or mutation of HLA-molecules (30–33), target antigens (34), or activating co-receptors resulting in induction of T-cell anergy. Peripheral tolerance mechanisms can also lead to clonal deletion and permanent loss of T cells recognizing abundantly expressed antigens in murine models (35). Parallel tracking of neoantigen-specific T cells and cognate tumor cells in an ovarian cancer patient with progressive disease showed that expansion of a tumor clone was accompanied by disappearance of the reactive T cells, suggesting deletional tolerance (9, 36). The possibility to therapeutically reverse immunosuppression has been demonstrated by checkpoint inhibition, which has led to impressive clinical responses in multiple cancer types, reviewed in Ref. (37). A large number of patients do, however, not respond, and the great majority of treated patients eventually relapse. Two recent papers have shed light on potential mechanisms for this.

Persistent stimulation by cognate antigen or exposure to inflammatory signals can lead to T cell exhaustion, which impairs T-cell effector functions. This dysfunctional state is characterized by an altered transcriptional program, including high expression of multiple inhibitory receptors such as programmed cell death 1 (PD-1). Recent studies indicate that exhausted T cells acquire an epigenetic profile that is distinct from that of effector and memory T cells and can only minimally be remodeled by PD-1 blockade therapy (38, 39). This epigenetic programming of T cells from a functional to dysfunctional state is suggested to occur in two phases, initially to a plastic state from which T cells can be rescued, and subsequently to a fixed dysfunctional state where T cells are resistant to reprogramming (40). This could possibly explain frequent clinical relapses following treatment with immune checkpoint inhibitors and has profound implications for the development of future immunotherapies. If the tumor-responsive T-cell repertoire of the patient is incapable of exerting lasting tumor control even if inhibitory signals are discontinued, new and innovative ways to activate the immune system are required.

## Broadening the Endogenous T-Cell Response to Neoantigens

Several studies have demonstrated that personal neoantigen vaccination protocols can elicit neoantigen-specific T-cell responses that are not detectable before vaccination (22–24), suggesting that insufficient priming partially accounts for limited neoantigen-specific T-cell responses in cancer patients. Two clinical studies pioneering mRNA-based (33) and peptide-based (24) personalized neoantigen vaccines in metastatic melanoma recently demonstrated clinical relevance of vaccination-induced responses. Thus, reduction in the frequencies of metastatic events (33) and direct recognition of tumor by some of the induced T-cell specificities (24, 33) were observed. T-cell responses to the majority of the vaccine antigens were *de novo* responses, supporting the view that the potential of the T-cell repertoire can be optimized by more effective priming. Such *de novo* responses are expected to mobilize naïve T cells that are not exhausted or dysfunctional. In the study by Ott et al., 16% of the peptides used for vaccination induced a CD8 response and 60% a CD4 response (24). Clinical response rates might, however, increase if an even higher number of verified CD8 epitopes could be included and a larger fraction of the induced T-cell specificities would translate into tumor-reactive responses (24). Thus, further studies to improve on antigen selection might be advantageous.

## Donor-Derived T Cells can Mediate Graft-Versus-Tumor Effects Following Allogeneic Hematopoietic Stem Cell Transplantation (alloHSCT)

In alloHSCT, donor-derived T cells can overcome the insufficiency of patient immunity. Alone or in combination with donor lymphocyte infusions (DLI), alloHSCT is frequently used to treat hematological cancers and still remains the only potentially curative treatment for many hematological malignancies [reviewed in Ref. (41)]. In an HLA-matched alloHSCT, the desired graft-versus-tumor reactivity (GvT) is thought to be mainly mediated by donor T cells recognizing peptides from polymorphic proteins, so called minor histocompatibility antigens (mHAg). These are generated by genetic differences between the donor and the host and presented by matched HLA on the malignant cells [reviewed in Ref. (42)]. Immunogenic mHAgs in the recipient are recognized by T cells from a donor lacking the immunogenic allele. mHAgs can be encoded by the Y chromosome or be autosomal. Autosomal mHAgs are most commonly derived from nonsynonymous single nucleotide polymorphisms, which result in single amino acid differences in the encoded proteins. Thus, mHAgs are seen as “neoantigens” by the donor T cells. Hematopoietic cells are preferentially recognized as they are more easily accessible than cells in solid tissues and they frequently express high levels of HLA class I and II, costimulatory receptors and adhesion molecules [reviewed in Ref. (42)]. However, donor T-cell reactivity to broadly expressed immunogenic mHAgs on healthy tissues bears the risk of potentially detrimental graft-versus-host disease (GvHD). DLIs can induce complete remissions in patients with relapsing leukemia after alloHSCT (43–46). The GvT effect is considered to be dependent on the presence of host antigen-presenting cells capable of efficiently displaying recipient’s hematopoietic lineage-restricted mHAgs to the donor T cells (47).

The powerful immune responses of GvT and GvHD demonstrate the ability of donor T cells to attack and kill defined cell types dependent on recognition of antigens differing between host and donor by a single amino acid. In fact, it is possible that tumor-specific neoantigens also serve as clinically relevant targets mediating GvT following alloHSCT. This could be suggested by the fact that syngeneic alloHSCT from a genetically identical twin can result in similar long-term disease-free survival rates as alloHSCT from an HLA-matched donor, but in the absence of allogeneic GvT (48–50). The relevance of neoantigens as targets for GvT was shown by a study in which two chronic lymphocytic leukemia patients with durable remission after alloHSCT were monitored for cytotoxic T-cell responses against predicted tumor-specific neoantigens and found to mount long-term responses against personal neoantigens derived from three different genes (25). As donor T cells have not been exposed to the peripheral tolerance mechanisms of the tumor, they can strongly recognize defined mHAgs or neoantigens presented by patient cancer cells. The possibility to specifically target donor T cells to patient neoantigens has, however, not been therapeutically explored thus far.

## Donor-Derived T-Cell Responses Reveal a High Frequency of Immunogenic Neoantigens

Advancements in sequencing techniques and computational sequence analysis tools have enabled fast identification of somatic mutations in expressed genes in individual tumors. The precision level of computer algorithms predicting potential neoepitopes recognized by T cells is, however, not known. A main challenge, therefore, remains to rapidly select among the large number of candidate neoantigens those that translate into clinically efficient immune responses. The uncompromised T-cell repertoires from HLA-matched donors hold an unrealized potential when addressing these challenges as they retain their inherent capability to respond to immunogenic neoantigens. Donor T-cell repertoires could thus be applied for identification of neoepitopes, independently of blood sampling from the patient. This was recently demonstrated by Strønen et al. by coculturing the non-adherent fraction of peripheral blood mononuclear cells with autologous monocyte-derived dendritic cells electroporated with *in vitro*-translated tandem minigene library RNA encoding potential neoepitopes from three melanoma patients, and by detecting neoantigen-responsive T cells with fluorescently labeled HLA-multimers. The study revealed that 11 out of 57 predicted HLA-A*02:01-binding neoepitopes were recognized by the nontolerized CD8 T-cell repertoires of healthy blood donors, 10 of which were ignored by the autologous TILs of the patient (15). Importantly, donor T-cell populations also recognized cognate neoepitopes when endogenously presented by the patient’s tumor cells, suggesting high-avidity T-cell responses. This study showed that a much higher frequency of neoantigens was immunogenic than was anticipated from analyzing the patient’s autologous *in vivo* T-cell responses. T-cell receptors (TCRs) isolated from the neoantigen-reactive donor T cells efficiently retargeted third party T cells to recognize patient-derived melanoma cells harboring the targeted mutations, suggesting that patient T cells redirected with neoantigen-targeted TCR could be effective in gene therapy (15).

## Donor-Derived Immunity to Design Personalized Immunotherapy

**Figure 1:**
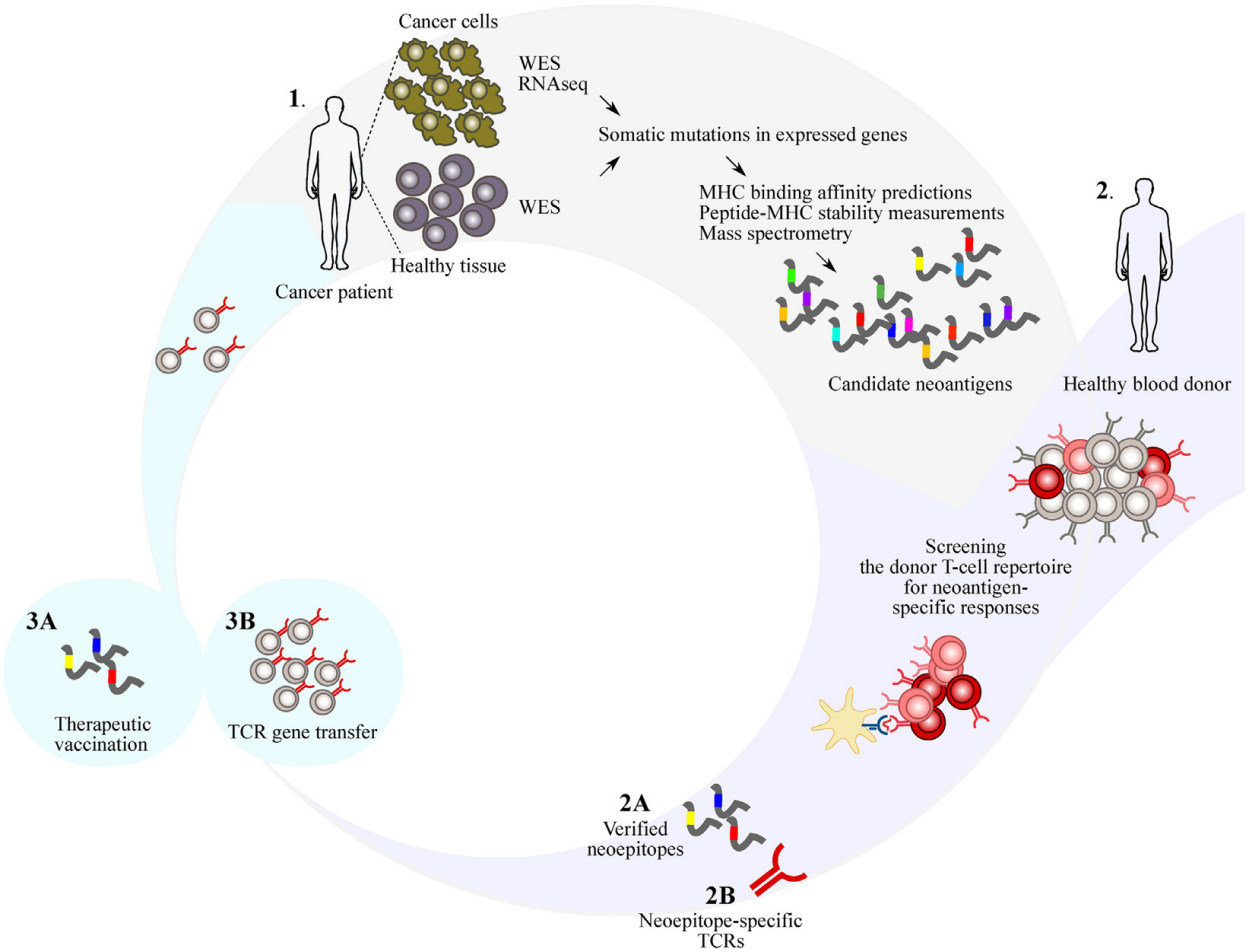
Identification of immunogenic neoantigens is the major technical challenge in genome-based personalized immunotherapy. **1**. Advances in sequencing techniques and computational sequence analysis tools have enabled rapid identification of somatic mutations in expressed genes that are capable of generating potential neoantigens. Human leukocyte antigen (HLA) binding affinity algorithms can narrow down the number of potential neoantigens, but are insufficient in predicting aspects contributing to immunogenicity. **2**. Nontolerized T-cell repertoires of healthy donors HLA-matched with the patient can be used to identify neoepitopes **(A)**, and T-cell receptors (TCRs) from the neoantigen-responsive donor T cells can be isolated **(B)**. **3**. The identified neoantigens can be used for therapeutic vaccination to (prime and) expand neoantigen-specific T cells in the patient repertoire **(A)**. Alternatively, the TCRs identified from the neoantigen-responsive donor T cells can be used to retarget patient T cells to recognize the tumor **(B)**. WES, whole-exome sequencing; RNAseq, RNA sequencing.

When the endogenous T-cell repertoire of the cancer patient is insufficient at controlling the disease, donor-derived immunity might provide rescue. One of the most successful examples is the adoptive transfer of T cells genetically modified by chimeric antigen receptors (CARs). T cells engineered to express CARs, harboring the antigen-recognition domain of an antibody grafted onto signaling domains that confer T-cell activation, can mediate selective killing of defined cell subsets. Treatment with CD19-targeted CAR T-cells consistently leads to complete response rates of 70–90% in acute lymphoblastic B-cell leukemia and has shown promise in non-Hodgkin B-cell lymphoma (51). The success of CAR T-cells to treat B-cell malignancies has, however, yet to be extended to other hematological cancers or solid tumors. A major obstacle is the identification of cell-type specific cell-surface molecules that can be safely targeted. In contrast to CARs, TCRs can recognize also intracellular antigens when presented in the context of surface MHC-molecules, opening the possibility to employ neoantigen-responsive TCRs to redirect patient T cells. TCRs from neoantigen-responsive donor T cells can be isolated and introduced into patient’s naïve or central memory T cells with high proliferative and functional potential. Adoptive transfer of genetically engineered T cells can create specificities not present or irreversibly inhibited in the patient, thus broadening the spectrum of the naturally occurring antitumor immunity (Figure 1). However, to make gene therapy with personalized, neoantigen-specific TCRs a feasible clinical option, the time required to identify clinically effective and safe TCRs has to be reduced. Furthermore, the cost and labor-intensive procedures associated with, and facilities required for, current retro- or lentiviral protocols for gene transfer are prohibiting widespread use of neoantigen-specific TCR gene therapy. Non-viral approaches for gene transfer might represent promising alternatives [discussed in Ref. (52)]. To this end, a recent study demonstrated efficient *in vivo* delivery of CAR genes using T-cell-targeted nanoparticles in mice, possibly representing a practical way to rapidly deliver genes (53).

A systematic screening for immunogenic neoantigens from the big pool of candidate epitopes using donor T-cell repertoires could advance our understanding of the rules determining immunogenicity. This could in turn enable development of more accurate prediction tools to identify neoepitopes. The need for sampling of blood from often heavily pretreated patients, and the use of patient T cells impaired by a variety of immunosuppressive mechanisms, would thus be circumvented. Proof of principle that such a screening with donor T-cell repertoires is possible was shown in Ref. (15), but the development of faster culture protocols and rapid identification of high-affinity TCRs would be desirable.

Screening for the ability of candidate neoantigenic peptides to induce responses in CD8 T cells from healthy, donor-derived T-cell repertoires was combined with a novel, flow cytometry-based assay to measure peptide–MHC stability (15). The results demonstrated that immunogenic neoantigens had a significantly longer half-life than the non-immunogenic ones. In fact, addition of measured peptide-HLA stability to predicted binding-affinity of the peptide to HLA significantly improved the precision levels by which the immunogenic peptides could be identified. These data corroborate well with previous studies of microbial peptides (54, 55), indicating that peptide–MHC stability is a better predictor of immunogenic peptides than peptide–MHC binding-affinity. Thus, development of assays which facilitate high-throughput stability measurements are called upon.

## Concluding Remarks

Tumors implement several immunosuppressive mechanisms to evade the immune defense of the cancer patient. These peripheral tolerance mechanisms can either reversibly or irreversibly impede the effector function of the patient’s tumor-targeting T-cell repertoire. Immunotherapies with expanded TILs or checkpoint blockade rely on specificities present in the patient’s own T-cell repertoire. Although clinical benefits are remarkable, they are often transient.

Transplantation of the immune system from an HLA-matched donor, which has not been compromised by tumor-induced peripheral tolerance, can induce cures in patients with hematological malignancies. The beneficial and powerful GvT effect of alloHSCT is mainly driven by donor T cells recognizing single amino acid differences in polymorphic peptides. However, since the targets are unknown and unpredictable, the desired GvT effect may be accompanied by potentially detrimental GvHD.

Personalized immunotherapies aim to explicitly target tumor-specific neoantigens, minimizing the risk of T-cell attack on healthy tissues. However, several hurdles have to be overcome to make genome-based approaches a treatment option for large groups of patients. Whole-exome sequencing can rapidly identify possible neoantigens in individual tumors, but defining those neoantigens that are immunogenic and clinically applicable remains a time-consuming, demanding task. Here, the unlimited source of donor T-cell repertoires can prove very informative. Identification of immunogenic neoantigens can guide the design of personalized vaccination and adoptive T-cell transfer therapies, and educate algorithms to become more accurate in predicting neoantigen immunogenicity. Neoantigen-reactive donor T cells can also provide TCRs, which can be used to retarget patient’s naïve T cells to attack the tumor. Simultaneous targeting of multiple neoantigens expressed homogeneously in the tumor and essential for maintaining the tumorigenic phenotype, thus unlikely to be lost, might be ideal to achieve durable clinical responses. Off-the-shelf TCRs targeting neoantigens derived from driver mutations recurrent in large patient groups and in prevalent tumor types would be most practical. Such neoantigens are, however, scarce and appear poorly immunogenic when presented on patient HLA (56). Thus, there is a high demand for strategies to rapidly define clinically applicable personal neoantigens, a challenge that potentially can be answered by donor T-cell repertoires.

## Author Contributions

Both authors made substantial intellectual contribution to the manuscript, and approved it for publication.

## Conflict of Interest Statement

JO is engaged in a research collaboration with Kite Pharma.

## Abbreviations

alloHSCT, allogeneic hematopoietic stem cell transplantation; CAR, chimeric antigen receptor; DLI, donor lymphocyte infusion; GvHD, graft versus host disease; GvT, graft versus tumor; HLA, human leukocyte antigen; mHAg, minor histocompatibility antigen; TCR, T-cell receptor; TIL, tumor-infiltrating lymphocyte.

## References

[1] Snyder A Makarov V Merghoub T Yuan J Zaretsky JM Desrichard A Genetic basis for clinical response to CTLA-4 blockade in melanoma. N Engl J Med (2014) 371(23):2189–99. 10.1056/NEJMoa1406498 25409260 PMC4315319

[2] Le DT Uram JN Wang H Bartlett BR Kemberling H Eyring AD PD-1 blockade in tumors with mismatch-repair deficiency. N Engl J Med (2015) 372(26):2509–20. 10.1056/NEJMoa1500596 26028255 PMC4481136

[3] Rizvi NA Hellmann MD Snyder A Kvistborg P Makarov V Havel JJ Cancer immunology. Mutational landscape determines sensitivity to PD-1 blockade in non-small cell lung cancer. Science (2015) 348(6230):124–8. 10.1126/science.aaa1348 25765070 PMC4993154

[4] Van Allen EM Miao D Schilling B Shukla SA Blank C Zimmer L Genomic correlates of response to CTLA-4 blockade in metastatic melanoma. Science (2015) 350(6257):207–11. 10.1126/science.aad0095 26359337 PMC5054517

[5] Le DT Durham JN Smith KN Wang H Bartlett BR Aulakh LK Mismatch-repair deficiency predicts response of solid tumors to PD-1 blockade. Science (2017) 357(6349):409–13. 10.1126/science.aan6733 28596308 PMC5576142

[6] van Rooij N van Buuren MM Philips D Velds A Toebes M Heemskerk B Tumor exome analysis reveals neoantigen-specific T-cell reactivity in an ipilimumab-responsive melanoma. J Clin Oncol (2013) 31(32):e439–42. 10.1200/JCO.2012.47.7521 24043743 PMC3836220

[7] Robbins PF Lu YC El-Gamil M Li YF Gross C Gartner J Mining exomic sequencing data to identify mutated antigens recognized by adoptively transferred tumor-reactive T cells. Nat Med (2013) 19(6):747–52. 10.1038/nm.3161 23644516 PMC3757932

[8] Lu YC Yao X Crystal JS Li YF El-Gamil M Gross C Efficient identification of mutated cancer antigens recognized by T cells associated with durable tumor regressions. Clin Cancer Res (2014) 20(13):3401–10. 10.1158/1078-0432.CCR-14-0433 24987109 PMC4083471

[9] Wick DA Webb JR Nielsen JS Martin SD Kroeger DR Milne K Surveillance of the tumor mutanome by T cells during progression from primary to recurrent ovarian cancer. Clin Cancer Res (2014) 20(5):1125–34. 10.1158/1078-0432.CCR-13-2147 24323902

[10] Tran E Turcotte S Gros A Robbins PF Lu YC Dudley ME Cancer immunotherapy based on mutation-specific CD4+ T cells in a patient with epithelial cancer. Science (2014) 344(6184):641–5. 10.1126/science.1251102 24812403 PMC6686185

[11] Tran E Ahmadzadeh M Lu YC Gros A Turcotte S Robbins PF Immunogenicity of somatic mutations in human gastrointestinal cancers. Science (2015) 350(6266):1387–90. 10.1126/science.aad1253 26516200 PMC7445892

[12] Cohen CJ Gartner JJ Horovitz-Fried M Shamalov K Trebska-McGowan K Bliskovsky VV Isolation of neoantigen-specific T cells from tumor and peripheral lymphocytes. J Clin Invest (2015) 125(10):3981–91. 10.1172/JCI82416 26389673 PMC4607110

[13] Linnemann C van Buuren MM Bies L Verdegaal EM Schotte R Calis JJ High-throughput epitope discovery reveals frequent recognition of neo-antigens by CD4+ T cells in human melanoma. Nat Med (2015) 21(1):81–5. 10.1038/nm.3773 25531942

[14] Gros A Parkhurst MR Tran E Pasetto A Robbins PF Ilyas S Prospective identification of neoantigen-specific lymphocytes in the peripheral blood of melanoma patients. Nat Med (2016) 22(4):433–8. 10.1038/nm.4051 26901407 PMC7446107

[15] Stronen E Toebes M Kelderman S van Buuren MM Yang W van Rooij N Targeting of cancer neoantigens with donor-derived T cell receptor repertoires. Science (2016) 352(6291):1337–41. 10.1126/science.aaf2288 27198675

[16] Prickett TD Crystal JS Cohen CJ Pasetto A Parkhurst MR Gartner JJ Durable complete response from metastatic melanoma after transfer of autologous T cells recognizing 10 mutated tumor antigens. Cancer Immunol Res (2016) 4(8):669–78. 10.1158/2326-6066.CIR-15-0215 27312342 PMC4970903

[17] Parkhurst M Gros A Pasetto A Prickett T Crystal JS Robbins P Isolation of T-cell receptors specifically reactive with mutated tumor-associated antigens from tumor-infiltrating lymphocytes based on CD137 expression. Clin Cancer Res (2016) 23(10):2491–505. 10.1158/1078-0432.CCR-16-2680 27827318 PMC6453117

[18] McGranahan N Furness AJ Rosenthal R Ramskov S Lyngaa R Saini SK Clonal neoantigens elicit T cell immunoreactivity and sensitivity to immune checkpoint blockade. Science (2016) 351(6280):1463–9. 10.1126/science.aaf1490 26940869 PMC4984254

[19] Kalaora S Barnea E Merhavi-Shoham E Qutob N Teer JK Shimony N Use of HLA peptidomics and whole exome sequencing to identify human immunogenic neo-antigens. Oncotarget (2016) 7(5):5110–7. 10.18632/oncotarget.6960 26819371 PMC4868674

[20] Bentzen AK Marquard AM Lyngaa R Saini SK Ramskov S Donia M Large-scale detection of antigen-specific T cells using peptide-MHC-I multimers labeled with DNA barcodes. Nat Biotechnol (2016) 34(10):1037–45. 10.1038/nbt.3662 27571370

[21] Bassani-Sternberg M Braunlein E Klar R Engleitner T Sinitcyn P Audehm S Direct identification of clinically relevant neoepitopes presented on native human melanoma tissue by mass spectrometry. Nat Commun (2016) 7:13404. 10.1038/ncomms13404 27869121 PMC5121339

[22] Carreno BM Magrini V Becker-Hapak M Kaabinejadian S Hundal J Petti AA Cancer immunotherapy. A dendritic cell vaccine increases the breadth and diversity of melanoma neoantigen-specific T cells. Science (2015) 348(6236):803–8. 10.1126/science.aaa3828 25837513 PMC4549796

[23] Li F Chen C Ju T Gao J Yan J Wang P Rapid tumor regression in an Asian lung cancer patient following personalized neo-epitope peptide vaccination. Oncoimmunology (2016) 5(12):e1238539. 10.1080/2162402X.2016.1238539 28123873 PMC5214696

[24] Ott PA Hu Z Keskin DB Shukla SA Sun J Bozym DJ An immunogenic personal neoantigen vaccine for patients with melanoma. Nature (2017) 547(7662):217–21. 10.1038/nature22991 28678778 PMC5577644

[25] Rajasagi M Shukla SA Fritsch EF Keskin DB DeLuca D Carmona E Systematic identification of personal tumor-specific neoantigens in chronic lymphocytic leukemia. Blood (2014) 124(3):453–62. 10.1182/blood-2014-04-567933 24891321 PMC4102716

[26] Alexandrov LB Nik-Zainal S Wedge DC Aparicio SA Behjati S Biankin AV Signatures of mutational processes in human cancer. Nature (2013) 500(7463):415–21. 10.1038/nature12477 23945592 PMC3776390

[27] Tran E Robbins PF Lu YC Prickett TD Gartner JJ Jia L T-cell transfer therapy targeting mutant KRAS in cancer. N Engl J Med (2016) 375(23):2255–62. 10.1056/NEJMoa1609279 27959684 PMC5178827

[28] Baitsch L Fuertes-Marraco SA Legat A Meyer C Speiser DE. The three main stumbling blocks for anticancer T cells. Trends Immunol (2012) 33(7):364–72. 10.1016/j.it.2012.02.006 22445288

[29] Koyama S Akbay EA Li YY Herter-Sprie GS Buczkowski KA Richards WG Adaptive resistance to therapeutic PD-1 blockade is associated with upregulation of alternative immune checkpoints. Nat Commun (2016) 7:10501. 10.1038/ncomms10501 26883990 PMC4757784

[30] Maeurer MJ Gollin SM Storkus WJ Swaney W Karbach J Martin D Tumor escape from immune recognition: loss of HLA-A2 melanoma cell surface expression is associated with a complex rearrangement of the short arm of chromosome 6. Clin Cancer Res (1996) 2(4):641–52. 9816214

[31] Shukla SA Rooney MS Rajasagi M Tiao G Dixon PM Lawrence MS Comprehensive analysis of cancer-associated somatic mutations in class I HLA genes. Nat Biotechnol (2015) 33(11):1152–8. 10.1038/nbt.3344 26372948 PMC4747795

[32] Zaretsky JM Garcia-Diaz A Shin DS Escuin-Ordinas H Hugo W Hu-Lieskovan S Mutations associated with acquired resistance to PD-1 blockade in melanoma. N Engl J Med (2016) 375(9):819–29. 10.1056/NEJMoa1604958 27433843 PMC5007206

[33] Sahin U Derhovanessian E Miller M Kloke BP Simon P Lower M Personalized RNA mutanome vaccines mobilize poly-specific therapeutic immunity against cancer. Nature (2017) 547(7662):222–6. 10.1038/nature23003 28678784

[34] Anagnostou V Smith KN Forde PM Niknafs N Bhattacharya R White J Evolution of neoantigen landscape during immune checkpoint blockade in non-small cell lung cancer. Cancer Discov (2017) 7(3):264–76. 10.1158/2159-8290.CD-16-0828 28031159 PMC5733805

[35] Bogen B. Peripheral T cell tolerance as a tumor escape mechanism: deletion of CD4+ T cells specific for a monoclonal immunoglobulin idiotype secreted by a plasmacytoma. Eur J Immunol (1996) 26(11):2671–9. 10.1002/eji.1830261119 8921954

[36] Martin SD Wick DA Nielsen JS Little N Holt RA Nelson BH A library-based screening method identifies neoantigen-reactive T cells in peripheral blood prior to relapse of ovarian cancer. Oncoimmunology (2017). 10.1080/2162402X.2017.1371895 PMC5739566 29296522

[37] Topalian SL Drake CG Pardoll DM. Immune checkpoint blockade: a common denominator approach to cancer therapy. Cancer Cell (2015) 27(4):450–61. 10.1016/j.ccell.2015.03.001 25858804 PMC4400238

[38] Pauken KE Sammons MA Odorizzi PM Manne S Godec J Khan O Epigenetic stability of exhausted T cells limits durability of reinvigoration by PD-1 blockade. Science (2016) 354(6316):1160–5. 10.1126/science.aaf2807 27789795 PMC5484795

[39] Sen DR Kaminski J Barnitz RA Kurachi M Gerdemann U Yates KB The epigenetic landscape of T cell exhaustion. Science (2016) 354(6316):1165–9. 10.1126/science.aae0491 27789799 PMC5497589

[40] Philip M Fairchild L Sun L Horste EL Camara S Shakiba M Chromatin states define tumour-specific T cell dysfunction and reprogramming. Nature (2017) 545(7655):452–6. 10.1038/nature22367 28514453 PMC5693219

[41] Singh AK McGuirk JP. Allogeneic stem cell transplantation: a historical and scientific overview. Cancer Res (2016) 76(22):6445–51. 10.1158/0008-5472.CAN-16-1311 27784742

[42] Falkenburg JH Jedema I. Allo-reactive T cells for the treatment of hematological malignancies. Mol Oncol (2015) 9(10):1894–903. 10.1016/j.molonc.2015.10.014 26578450 PMC5528734

[43] Collins RH Jr Shpilberg O Drobyski WR Porter DL Giralt S Champlin R Donor leukocyte infusions in 140 patients with relapsed malignancy after allogeneic bone marrow transplantation. J Clin Oncol (1997) 15(2):433–44. 10.1200/JCO.1997.15.2.433 9053463

[44] Falkenburg JH Wafelman AR Joosten P Smit WM van Bergen CA Bongaerts R Complete remission of accelerated phase chronic myeloid leukemia by treatment with leukemia-reactive cytotoxic T lymphocytes. Blood (1999) 94(4):1201–8. 10438707

[45] Porter DL Collins RH Jr Hardy C Kernan NA Drobyski WR Giralt S Treatment of relapsed leukemia after unrelated donor marrow transplantation with unrelated donor leukocyte infusions. Blood (2000) 95(4):1214–21. 10666193

[46] Deol A Lum LG. Role of donor lymphocyte infusions in relapsed hematological malignancies after stem cell transplantation revisited. Cancer Treat Rev (2010) 36(7):528–38. 10.1016/j.ctrv.2010.03.004 20381970 PMC2921454

[47] Mapara MY Kim YM Wang SP Bronson R Sachs DH Sykes M. Donor lymphocyte infusions mediate superior graft-versus-leukemia effects in mixed compared to fully allogeneic chimeras: a critical role for host antigen-presenting cells. Blood (2002) 100(5):1903–9. 10.1182/blood-2002-01-0023 12176915

[48] Bierman PJ Sweetenham JW Loberiza FR Jr Taghipour G Lazarus HM Rizzo JD Syngeneic hematopoietic stem-cell transplantation for non-Hodgkin’s lymphoma: a comparison with allogeneic and autologous transplantation – the lymphoma working committee of the international bone marrow transplant registry and the European group for blood and marrow transplantation. J Clin Oncol (2003) 21(20):3744–53. 10.1200/JCO.2003.08.054 12963703

[49] Kroger N Brand R van Biezen A Bron D Blaise D Hellstrom-Lindberg E Stem cell transplantation from identical twins in patients with myelodysplastic syndromes. Bone Marrow Transplant (2005) 35(1):37–43. 10.1038/sj.bmt.1704701 15531907

[50] Pavletic SZ Zhou G Sobocinski K Marti G Doney K DiPersio J Genetically identical twin transplantation for chronic lymphocytic leukemia. Leukemia (2007) 21(12):2452–5. 10.1038/sj.leu.2404928 17728782

[51] Khalil DN Smith EL Brentjens RJ Wolchok JD. The future of cancer treatment: immunomodulation, CARs and combination immunotherapy. Nat Rev Clin Oncol (2016) 13(5):273–90. 10.1038/nrclinonc.2016.25 26977780 PMC5551685

[52] Olweus J Manufacture of CAR-T cells in the body. Nat Biotechnol (2017) 35(6):520–1. 10.1038/nbt.3898 28591119

[53] Smith TT Stephan SB Moffett HF McKnight LE Ji W Reiman D In situ programming of leukaemia-specific T cells using synthetic DNA nanocarriers. Nat Nanotechnol (2017) 12(8):813–20. 10.1038/nnano.2017.57 28416815 PMC5646367

[54] van der Burg SH Visseren MJ Brandt RM Kast WM Melief CJ. Immunogenicity of peptides bound to MHC class I molecules depends on the MHC-peptide complex stability. J Immunol (1996) 156(9):3308–14. 8617954

[55] Harndahl M Rasmussen M Roder G Dalgaard Pedersen I Sorensen M Nielsen M Peptide-MHC class I stability is a better predictor than peptide affinity of CTL immunogenicity. Eur J Immunol (2012) 42(6):1405–16. 10.1002/eji.201141774 22678897

[56] Marty R Kaabinejadian S Rossell D Slifker MJ van de Haar J Engin HB MHC-I genotype restricts the oncogenic mutational landscape. Cell (2017). 10.1016/j.cell.2017.09.050 29107334 PMC5711564

